# The Role of Herbal Medicine in the Management of Oral Conditions: A Comprehensive Review 

**DOI:** 10.30476/dentjods.2025.106062.2629

**Published:** 2026-06-01

**Authors:** Mehrdad Kolahi, Mohammad Sobootkar, Sarah Hamidzade, Kimia Ghods

**Affiliations:** 1 Dental Student, Membership of Dental Material Research Center, Tehran Medical Sciences, Islamic Azad University, Tehran, Iran.; 2 Membership of Dental Material Research Center, Tehran Medical Sciences, Islamic Azad University, Tehran, Iran.

**Keywords:** Herbal Medicine, Oral Disorder, Complementary Therapies, Treatment Outcome, Drug Effects

## Abstract

Herbal medicine has been traditionally used in managing various health conditions, including oral diseases. With growing interest in natural treatments, numerous plant-derived compounds have been studied for their antimicrobial, anti-inflammatory, analgesic, and wound-healing properties. The present study aims to evaluate the influence of various types of herbal medicine in the management of oral conditions, along with their advantages and disadvantages. For this review article, a complete query was carried out on PubMed, Google Scholar, Embase, and Scopus databases, and the studies published during 2015-2024 were collected using the keywords such as "Herbal Medicine," "Oral Disorder," "Complementary Therapies", "Treatment Outcome," and "Drug Effects." After applying appropriate inclusion and exclusion criteria, 64 relevant articles focused on the application of herbal medicine in the treatment of oral diseases were selected and evaluated. Among all the medical herbs, aloe vera, turmeric, ginger, and green tea have shown more promising antimicrobial and anti-inflammatory properties that contribute to oral health. In other words, studies suggest that the mentioned herbs may aid in reducing plaque formation, managing gingival inflammation, promoting wound healing, and controlling oral microbial infections better than other herbs. As evidenced by the obtained results, herbal medicine holds promise as a complementary or alternative approach in oral care due to its bioactive properties and potential therapeutic benefits. However, further clinical trials and scientific validation is necessary to ensure safety, efficacy, and standardization before herbal treatments can be fully integrated into modern practice.

## Introduction

Since the dawn of civilization, humanity has been looking for ways to prevent and treat diseases. In this direction, it seems that the use of herbal medicines can play a notable role in improving the health of human society. Recently, the use of these plants alongside modern synthetic drugs has been on the rise. Therefore, the world health organization emphasizes the role of oral conditions in enhancing quality of life and the expanding tendency to use herbal medicines to enhance oral health [ 1 - 2 ].

Herbal medicines, due to their availability, lower cost, and reduced side effects, offer a logical and cost-effective alternative to synthetic drugs for managing oral health. The side effects of synthetic drugs include tissue irritation, allergic reactions, and gastrointestinal issues, which can negatively impact a patient's quality of life. Nonetheless, one other significant problem with synthetic drugs is the phenomenon of bacterial resistance to antibiotics [ 2 ]. 

The overuse and misuse of antibiotics have led to bacterial resistance, making infections more difficult to treat. This underscores the need to find more effective and safer alternatives. In this context, plant-based products with antimicrobial properties can serve as viable alternatives. Recent studies indicate that these medicinal plants can be effective in preventing and treating oral diseases through various mechanisms. These mechanisms include inhibiting bacterial growth, preventing their adhesion to surfaces, and reducing the production of acids and polysaccharides [ 3 - 4 ].

Despite the beneficial properties of medicinal plants in oral treatments highlighted in studies, there are also observed side effects that challenge their widespread application. Specifically, some medicinal plants may interact with medications taken by patients or dental materials. Furthermore, in some cases, these medications may even cause allergic reactions in certain individuals. Therefore, further clinical research is necessary to determine the efficacy and safety of herbal treatments for oral conditions [ 4 - 5 ]. Hence, this review study examines the diverse applications of herbal medicines in promoting oral health, along with their advantages and disadvantages.

## Materials and Method

This comprehensive review literature adhered to the preferred reporting items for systematic reviews and meta-analyses guidelines [ 6 ] to ensure a transparent search strategy. The search was limited to peer-reviewed journal articles published in English, excluding books, editorials, commentaries, dissertations, unpublished materials, and letters to the editor. PubMed, Google Scholar, Embase, and Scopus databases were used to identify relevant studies using variations of key terms. The key terms were carefully chosen to align with the study's topic and subsequently were refined by Boolean operators. The complete search strategies, customized for each database (PubMed, Embase, Scopus, Google Scholar), are available in Appendix 1.

**Appendix 1:** Complete Search Strategies

**PubMed**
("Phytotherapy"[MeSH] OR "Herbal Medicine" [MeSH]) AND ("Mouth Diseases"[MeSH] OR "Oral Disorder" [MeSH]) AND ("Complementary Therapies" [MeSH]) AND ("Therapeutics"[MeSH] OR "Treatment Outcome"[MeSH] OR "Disease Management"[MeSH]) AND ("Drug Effects"[MeSH] OR "Pharmacology" [MeSH] OR "Plant Extracts/pharmacology"[MeSH]) AND ("Oral cancer" OR "Mouth neoplasms"[MeSH] OR "Oral submucous fibrosis"[MeSH] OR "candidiasis, oral" [MeSH] OR "mouth ulcers"[MeSH] OR "oral mucositis" OR "stomatitis"[MeSH] OR "stomatitis, aphthous"[MeSH] OR "herpes simplex"[MeSH] OR "Oral Lichen Planus"[Mesh] OR "burning mouth syndrome" [MeSH] OR "halitosis"[MeSH] OR "xerostomia" [MeSH]) AND ("aloe vera" OR "aloe"[MeSH] OR "ginger" OR "zingiber officinale"[MeSH] OR "green tea" OR "camellia sinensis"[MeSH] OR "turmeric" OR "curcuma"[MeSH] OR "garlic" OR "allium sativum" [MeSH] OR "triphala" OR "licorice" OR "thyme" OR "capsaicin" OR "cinnamon" OR "berberine" OR "psidium guajava" OR "chamomile" OR "pomegranate" OR "cranberry" OR "siwak" OR "Chinese flavonoid herbs" OR "strawberry" OR "herbal paste")
**Embase**
('phytotherapy'/exp OR 'herbal medicine'/exp) AND ('mouth disease'/exp OR 'oral disorder'/exp) AND ('Complementary Therapies'/exp) AND ('therapy'/exp OR 'treatment outcome'/exp OR 'disease management'/ exp) AND ('drug effect'/exp OR 'pharmacology'/exp OR 'plant extract'/exp) AND ('oral cancer'/exp OR 'mouth tumor'/exp OR 'oral submucous fibrosis'/exp OR 'oral candidiasis'/exp OR 'mouth ulcer'/exp OR 'oral mucositis'/exp OR 'stomatitis'/exp OR 'aphthous stomatitis'/ exp OR 'herpes simplex'/exp OR 'oral lichen planus'/exp OR 'burning mouth syndrome'/exp OR 'halitosis'/ exp OR 'xerostomia'/exp) AND ('aloe vera'/exp OR 'aloe'/exp OR 'ginger'/exp OR 'zingiber officinale'/ exp OR 'green tea'/exp OR 'camellia sinensis'/exp OR 'turmeric'/exp OR 'curcuma'/exp OR 'garlic'/exp OR 'allium sativum' /exp OR 'triphala' OR 'licorice'/exp OR 'thyme'/ exp OR 'capsaicin'/exp OR 'cinnamon'/exp OR 'berberine'/exp OR 'psidium guajava'/exp OR 'chamomile'/exp OR 'pomegranate'/exp OR 'cranberry'/exp OR 'siwak' OR 'Chinese flavonoid herbs' OR 'strawberry' OR 'herbal paste')
**Scopus**
TITLE-ABS-KEY(("phytotherapy" OR "herbal medicine") AND ("mouth diseases" OR "oral disorder") AND ("Complementary Therapies") AND ("therapeutics" OR "treatment outcome" OR "disease management") AND ("drug effects" OR "pharmacology" OR "plant extracts") AND ("oral cancer" OR "mouth neoplasms" OR " oral submucous fibrosis" OR "oral candidiasis" OR "mouth ulcers" OR "oral mucositis" OR "stomatitis" OR "aphthous stomatitis" OR "herpes simplex" OR "oral lichen planus" OR "burning mouth syndrome" OR "halitosis" OR "xerostomia") AND ("aloevera" OR "aloe" OR "ginger" OR "zingier officinale" OR "green tea" OR "camellia sinensis" OR "turmeric" OR "curcuma" OR "garlic" OR "allium sativum" OR "triphala" OR "licorice" OR "thyme" OR "capsaicin" OR "cinnamon" OR "berberine" OR "psidium guajava" OR "chamomilee" OR "pomegranate" OR "cranberry" OR "siwak" OR "Chinese flavonoid herbs" OR "strawberry" OR "herbal paste").
**Google Scholar**
All in title: ("phytotherapy" OR "herbal medicine") AND ("oral diseases" OR "oral disorders") AND ("Complementary Therapies") AND ("aloe vera" OR "turmeric" OR "garlic" OR "green tea" OR "ginger" OR "triphala" OR "licorice")

In the screening phase, first, the Rayyan software was used to remove duplicates, and 1032 articles were excluded. Subsequently, all the articles were evaluated based on the topic of the article and subject of interest, the keywords, and the inclusion and exclusion criteria by all authors. The inclusion criteria in this research were (1) articles published in or after 2015, (2) articles written in English, and (3) articles focused on the use of herbal medicine or plant-based therapies in the prevention or treatment of oral diseases. The exclusion criteria included articles with unrelated information and topics, articles published before 2015, case reports, books, editorials, commentaries, dissertations, unpublished materials, and letters to the editor. It should be mentioned that the search was limited to studies published after 2015 to prioritize recent evidence reflecting current advancements in herbal therapies for oral diseases. Ultimately, according to the mentioned criteria and keywords, 1323 English-written articles were selected and retrieved. Since the full text of 541 articles was unavailable, the mentioned studies were accordingly removed.

**Figure 1:**
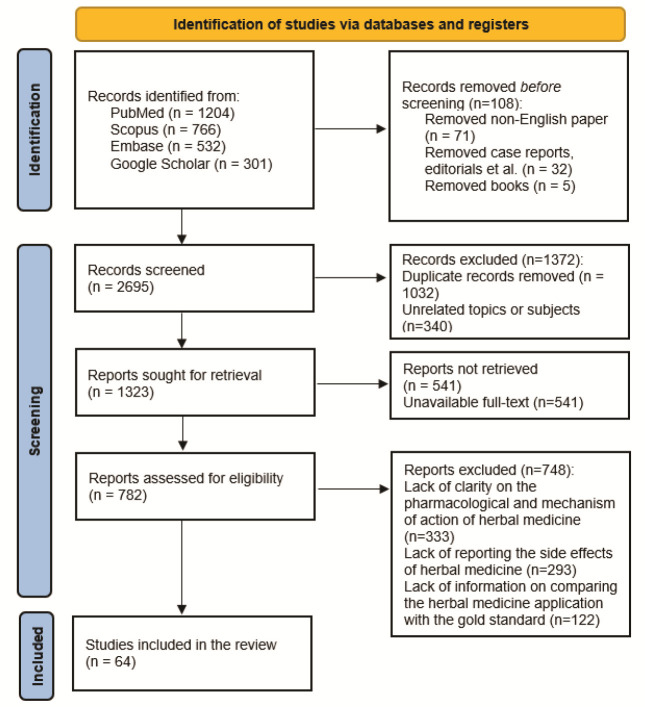
Methodology of the study

After retrieving articles, the evaluation of the eligibility of studies was conducted by all authors. In the beginning, a specialist in the field of herbal medicine (A. A) investigated the accuracy and reliability of data regarding herbal medicine. During this phase, 333 articles were excluded due to insufficient clarity regarding the pharmacological effects and mechanisms of action of the studied herbal medicines. In the next phase, the other authors evaluated the validity of the information presented in the articles using specific tools, the method of study, and the accordance of the studies 'results with the aims of the present research. After this precise review, 293 articles were excluded based on the absence of any information regarding the possible side effects of herbal medicine in oral treatments. Moreover, 122 articles were excluded due to a lack of information on comparing the herbal medicine application with the gold standard method in the management of oral conditions. Ultimately, 64 articles entered the study. In Figure 1, the study's methodology is presented. 

### Bias assessment

Two reviewers (MK and KG) independently reviewed and analyzed the risk of bias using appropriate tools based on study design. These tools included the Cochrane ROB-2 tool for randomized controlled trials, the Newcastle-Ottawa scale for non-randomized and observational studies, the SYRCLE’s risk of bias tool for *in vitro* and preclinical studies, and the AMSTAR-2 checklist for systematic reviews. 

## Results

The search strategy resulted in a selection of 64 articles, including 39 from PubMed, 12 from Scopus, 7 from Embase, and 6 from Google Scholar. After a comprehensive review of the literature, it was observed that 11 articles were focused on the overall concept of herbal medicine and its mechanism of action. Subsequently, 53 other articles illustrated the application of herbal medicine in the management of different oral conditions. Out of the 53 articles assessing herbal medicine' application in oral medicine, 10 articles were regarding oral cancers, 3 articles were about oral submucous fibrosis (OSMF), 10 articles were related to oral candidiasis, 4 articles were regarding oral ulcers, 4 articles were focused on oral mucositis, 1 article was related to recurrent aphthous stomatitis (RAS), 2 articles were about herpes simplex, 5 articles were regarding oral lichen planus (OLP), 2 articles were related to burning mouth syndrome (BMS ), 6 articles were about halitosis, and ultimately 6 articles discussed the application of herbal medicine in dry mouth.

### Risk of Bias Assessment 

The risk of bias assessment across included studies revealed varying levels of methodological quality. Among the 40 randomized controlled trials, 32% demonstrated low risk in randomization and allocation procedures, while 46% had some concerns (medium risk), and 22% were at high risk. Furthermore, blinding of participants and outcome assessors showed a lower proportion of studies at low risk (24%), with equal proportions (38%) presenting medium or high risk. In 16 observational studies, 41% were deemed to have low risk of confounding, 35% had moderate risk, and 24% were assessed as high risk. For five in vitro studies, 60% met criteria for low risk of bias, while 20% each showed moderate and high risk, indicating variability in methodological rigor across study types. Ultimately, among the three included systematic reviews, two were rated as “High” quality according to the AMSTAR-2 checklist, indicating a generally robust methodological standard. 

## Literature Review

Since ancient times, civilizations such as Egypt, China, and India have used medicinal plants for the prevention and management of oral diseases. However, over time, with the advent of scientific advancements and the production of synthetic drugs, the use of medicinal plants declined. In recent decades, interest in medicinal plants has resurged due to their fewer side effects compared to conventional synthetic drugs [ 7 ]. It has been observed that the use of herbal medicines allows patients to avoid many of the side effects typically associated with traditional medications. As a result, various studies have investigated the effects of herbal medicines on different oral diseases [ 8 - 9 ].

Oral diseases are among the significant global health challenges, encompassing a wide range of conditions that are now being managed using herbal medicines [ 10 - 12 ]. Among various oral diseases, the efficacy of herbal medicine on oral conditions such as oral cancer, OSMF, fungal infections caused by *Candida albicans* (*C.albicans*), oral ulcers, oral mucositis, RAS, oral herpes, OLP, BMS, dry mouth, and halitosis is discussed below. 

### Oral Cancer

Oral cancer is recognized as a critical global public health issue that requires prompt identification and treatment. One of its most common types is squamous cell carcinoma, which can develop in various parts of the oral cavity, including the lips, tongue, gums, floor of the mouth, buccal mucosa, and the oropharynx. Due to the extensive areas affected by squamous cell carcinoma, this disease necessitates comprehensive and multifaceted treatment approaches to minimize the side effects of therapies [ 13 - 14 ]. 

The standard treatment for oral cancer usually includes surgery, radiotherapy, and chemotherapy. These treatments are generally effective according to their precise targeting of cancerous tissues. However, standard treatments are accompanied by serious side effects such as nausea, vomiting, loss of appetite, diarrhea, mucositis, and oral numbness. These side effects can significantly impact patients' quality of life and complicate the treatment process. Consequently, herbal medicines have been proposed for disease management and pain relief [ 14 - 15 ].

Studies have shown that natural compounds extracted from plants can effectively inhibit the growth of cancer cells and protect normal cells from damage. Specifically, nuclear factor kappa B (NF-κB) and activator protein 1 (AP-1), which regulate genes associated with angiogenesis and the aggressive growth of cancer cells, are inhibited by natural plant compounds, leading to apoptosis [ 7 , 13 - 14 ].

Moreover, some studies have confirmed that Chinese herbal medicines have limited adverse effects on the treatment of oral cancers. In other words, natural compounds in Chinese herbal medicine, particularly flavonoids, offer a promising approach to targeting specific genetic mutations in oral cancer cells [ 7 , 16 ]. According to a study by Y. C. Huang *et al*. [ 7 ], Chinese herbal compounds containing flavonoids induce apoptosis through various mechanisms. These mechanisms include caspase activation, inhibition of anti-apoptotic proteins such as Bcl-2 and Bcl-xL, and modulation of signaling pathways like NF-κB, PI3K/AKT, and mTOR. Notably, these compounds exhibit no toxicity to normal cells, indicating a favorable therapeutic window. The results suggest that this approach may reduce the risks associated with oral cancer treatment and pave the way for innovative adjuvant therapies [ 7 ].

It is hypothesized that natural compounds found in medicinal plants may enhance the efficacy of synthetic drugs through synergistic interactions, potentially leading to improved therapeutic outcomes. This synergy can occur via multiple mechanisms, such as enhancing drug bioavailability, modulating pharmacokinetic properties, reducing drug resistance, or targeting complementary biological pathways [ 7 , 13 - 14 ]. However, potential risks associated with the concurrent use of chemotherapy drugs and Chinese herbal medicines containing flavonoids have been observed. These risks depend on interactions between chemotherapy drugs and Chinese herbal medicines, where the latter may interfere with anti-cancer drugs that metabolize enzymes and transporters [ 7 ].

In addition to Chinese herbal medicines, strawberries and turmeric are two plants with properties that play a significant role in improving oral health. Strawberries, due to their high vitamin C content and antioxidants, can reduce the colonization of *Streptococcus mutans*. The anti-cancer properties of strawberries prevent cellular mutations and reduce the risk of oral cells turning cancerous. On the other hand, turmeric, with its active compound curcumin, is known for its potent anti-inflammatory and anti-cancer properties. Nevertheless, curcumin can inhibit the cell cycle of cancer cells and induce apoptosis, making it highly effective in preventing the progression of oral cancer [ 7 , 17 ].

Furthermore, studies have shown that *Triphala*, an extract derived from the fruits of three plants (*Emblica officinalis, Terminalia bellirica*, and *Terminalia chebula*), has a remarkable anti-tumor influence on oral cancer cells. Specifically, *Triphala* can reduce the proliferative capacity of cancer cells, prevent their migration and invasion into surrounding tissues, induce cancer cell death, and exert its anti-tumor effects by influencing specific cell signaling pathways [ 18 - 19 ]. Supporting these findings, a study by S. Hu *et al*. [ 18 ] on zebrafish embryos demonstrated that *Triphala* effectively inhibits tumor growth and metastasis. In this study, various concentrations of *Triphala* (0, 125, 250, 500, 1000, and 2000µg/mL) were used to treat zebrafish embryos, and the maximum therapeutic concentration or minimum toxic concentration for anti-tumor effects was determined to be 500µg/mL [ 18 ].

Pomegranate (*Punica granatum*) is another plant known for its antioxidant and anti-inflammatory properties, which are effective in preventing oral cancers and improving gum health. Numerous studies have shown that pomegranate peel extract reduces the proliferation of oral cancer cells, such as HSC-2, HSC-3, HSC-4, and Ca9-22, thereby contributing to oral health [ 20 - 22 ].

Emerging evidence highlights the therapeutic potential of natural compounds, such as flavonoids in Chinese herbal medicine, curcumin in turmeric, ellagic acid in strawberries, and bioactive agents in *Triphala* and pomegranate. In fact, these compounds selectively target cancer cells via apoptosis induction, NF-κB inhibition, and PI3K/Akt pathway modulation. Moreover, these botanicals not only demonstrate anti-tumor efficacy but also exhibit minimal toxicity to healthy tissues, offering a promising adjunct to traditional therapies. While synergistic combinations with synthetic drugs show potential, careful consideration of herb-drug interactions is essential. Future research should focus on standardizing these natural therapies and integrating them into multimodal treatment regimens to improve outcomes for oral cancer patients [ 7 , 13 - 22 ].

### Oral Submucous Fibrosis (OSMF)

OSMF is a chronic and potentially malignant disorder predominantly observed in Asian countries. This condition is characterized by the hardening of the oral mucosa and the formation of fibrous bands in the submucosal layers, leading to difficulties in opening the mouth. It is particularly associated with habits such as chewing tobacco and areca nuts, and increases the risk of oral cancer [ 8 , 23 ].

Recently, physicians have turned to plant-derived formulations instead of conventional intralesional steroids for managing OSMF. A study investigated the efficacy of a new herbal paste formulation consisting of 10 grams of turmeric powder and 10 grams of Tulsi powder mixed in 10 mL of honey compared to a placebo containing antioxidants [ 8 ]. This research found that this combination, when used four times daily over three months, improved mouth opening and reduced tongue protrusion, burning sensation, whitening of the mucous membrane, and palpable fibrous bands [ 8 ]. This suggests that this paste formulation can serve as an effective and non-invasive treatment for OSMF [ 8 , 24 ].

Another recent study demonstrated that *Triphala* extract could mitigate the adverse effects of arecoline on oral epithelial cells and reduce the likelihood of OSMF [ 25 ]. In this study, *Triphala* at a concentration of 5µg/mL significantly reversed the aging and cell cycle arrest induced by arecoline, reducing the activity of beta-galactosidase which is an enzyme abundant in senescent cells. Additionally, *Triphala* decreased the expression of aging-related genes such as p16 and p21. These results indicate that *Triphala* treatment can help restore epithelial thickness in patients with OSMF and may be considered a complementary therapy to synthetic drugs [ 25 ].

Recent studies [ 8 , 23 - 25 ] demonstrate that formulations like turmeric, Tulsi, and honey paste improve mouth opening and reduce symptoms, while *Triphala* counteracts arecoline-induced epithelial damage by modulating senescence markers (p16, p21) and restoring cellular homeostasis. These natural therapies offer minimally invasive, adjunctive options with fewer side effects, addressing both symptom management and disease progression. Future research should focus on standardizing bioactive concentrations and evaluating long-term outcomes to integrate these botanicals into mainstream OSMF therapy protocols [ 8 , 23 - 25 ].

### Oral Candidiasis

Oral candidiasis caused by *C.albicans* is a common type of oral mucosal disease, particularly prevalent among denture wearers. Given the resistance of the fungus to traditional medications such as nystatin and clotrimazole, research has shifted toward the use of natural compounds as alternative treatments [ 10 , 26 ].

Garlic (*Allium sativum*) is one of the natural compounds that various studies have emphasized its therapeutic effects in improving oral candidiasis [ 27 - 28 ]. For instance, in a randomized clinical trial, an aqueous garlic solution at a concentration of 40 mg/mL was used three times daily for four weeks [ 27 ]. In another study, garlic paste (applied in sufficient quantity to cover the lesion entirely) along with a drop of 2% lidocaine gel, was administered four times daily for two weeks [ 28 ]. Both studies found that garlic, in both aqueous solution and paste forms, could reduce candidiasis symptoms with fewer side effects compared to nystatin [ 27 , 28 ].

Thyme is another herbal compound used to reduce various forms of oral candidiasis [ 29 ]. For example, in a study by Aghajani *et al*. [ 30 ], a spray containing thyme extract at a concentration of 25µL/mL and a spray containing 1% sodium hypochlorite were used 8 to 10 times to disinfect dentures contaminated with *C.albicans* In this study, dentures were first rinsed with water for 15 seconds, and after 10 minutes and 1 hour, they were rinsed with thyme spray in the control group and sodium hypochlorite in the placebo group. The results indicated that the antifungal effects of the sodium hypochlorite were superior to thyme extract within 10 minutes, but no significant difference was observed between the two groups over a longer period (1 hour) [ 30 ].

In addition to garlic and thyme, capsaicin, the active compound in chili peppers, is also used to improve oral candidiasis [ 31 ]. A recent study found that capsaicin at a high concentration of 95% can prevent the formation of *C.albicans* hyphae and disrupt ergosterol biosynthesis. Moreover, the synergistic effect of capsaicin with fluconazole may reduce the dosage of antifungal drugs, helping to avoid drug resistance and side effects [ 32 ].

Another widely used plant in dentistry is licorice, derived from the root of *Glycyrrhiza glabra*, whose pharmacological effects are attributed to its secondary components. Studies have shown that licorice extracts and their active compounds are beneficial in treating dental caries, gingivitis, periodontitis, candidiasis, and oral ulcers, with positive effects on oral microbial pathogens and immune responses [ 33 - 36 ]. In one study, a dose of 200 mg/kg of licorice extract was administered orally twice daily for five days to mice. The results showed that this compound significantly reduced infection and was effective against *C.albicans* compared to the control group [ 27 ].

*Cinnamomum zeylanicum* is another effective herbal compound being utilized against *C.albicans*. This herb is known for its antiseptic, antimicrobial, and analgesic properties. In a clinical study, a mouthwash containing 635 µg/mL of cinnamon was used for 60 seconds, three times daily for 15 days. The results demonstrated that this compound significantly reduced the symptoms of denture stomatitis [ 27 ].

Berberine, derived from medicinal plants, is recognized for its antimicrobial, antifungal, and antiviral properties which make it a potential treatment for oral candidiasis. In a study, doses of 1.95 to 250mg/L of berberine, 0.125 to 4mg/L of miconazole, and 0.5 to 16mg/L of fluconazole were administered orally and in combination with each other. The results showed that berberine alone had stronger antifungal effects compared to the two synthetic drugs [ 27 ].

Ginger, like the other herbal compounds mentioned, possesses antioxidant and antimicrobial properties and is known as a natural remedy for nausea and motion disorders. In one study, ginger concentrations ranging from 0.625 to 80mg/mL were used as a mouthwash for 24 hours, and the results showed a significant reduction in *C.albicans* biofilm formation at concentrations of 0.625 to 5mg/mL [ 27 ].

Emerging plant-based therapies, including garlic, thyme, capsaicin, licorice, cinnamon, berberine, and ginger, demonstrate potent antifungal activity against oral candidiasis. This outcome is probably due to targeting biofilm formation by herbal medicines. These herbs also offer advantages like reduced toxicity, immune modulation, and synergy with conventional antifungals to combat drug resistance. The observed results suggest natural components as promising adjuncts or alternatives for managing candida infections, particularly in azole-resistant or recurrent cases [ 10 , 26 - 36 ].

### Traumatic Oral Ulcers

Oral ulcers are among the most common mucosal problems in the oral cavity, caused by various factors. These factors are consuming acidic or spicy foods, hot objects or foods, accidental biting, orthodontic braces, dentures, ingredients in toothpaste, and certain medications. Although various synthetic drugs in the form of pastes, mouthwashes, tablets, patches, chewing gum, and gels have been proposed to alleviate the pain and discomfort caused by oral ulcers, the use of herbal medicines as alternatives has gained popularity in recent years [ 37 ].

*Psidium guajava* is an evergreen shrub whose alkaloids, carotenoids, phenols, and flavonoids, particularly quercetin, exhibit antibacterial and anti-ulcer properties. Accordingly, a recent study was performed that supported the positive anti-ulcer effects of *Psidium guajava*. This research demonstrated that a gel containing 100 grams of powdered *Psidium guajava* leaves effectively reduced the levels of *Staphylococcus aureus* and *E. coli* bacteria, leading to a shorter healing period for oral ulcers [ 38 ].

In addition to *Psidium guajava*, turmeric and its extract (*Curcuma longa*) have also been found effective in improving oral ulcers due to their anti-inflammatory and anti-arthritic properties. A laboratory study showed that 0.5 grams of a gel containing *Psidium guajava*, turmeric, ethanol, and distilled water effectively inhibited the growth of *C.albicans* in culture media. As a result, the combination of *Psidium guajava* and turmeric is now recognized as one of the strongest and most effective herbal remedies for improving oral ulcers [ 39 ].

Another herbal substance used for the treatment of chronic and traumatic ulcers is aloe vera. In a clinical trial study, a gel containing 80% aloe vera was found to significantly prevent or improve traumatic ulcers caused by fixed orthodontic appliances within one month of application. In this study, the gel was applied twice daily for 15 minutes with gentle massage on the ulcers [ 40 ].

Herbal medicines like *Psidium guajava*, turmeric, and aloe vera offer effective, natural alternatives for treating oral ulcers by reducing bacterial load (*S. aureus*, *E. coli*), inhibiting *C.albicans* growth, and accelerating wound healing through anti-inflammatory and antimicrobial properties. This outcome is supported by clinical studies supporting emphasizing their higher efficacy in gel formulations for both traumatic and recurrent ulcer management [ 37 - 40 ].

### Oral Mucositis

Oral mucositis is an inflammatory mucosal injury characterized by erythema or ulceration of the oral mucosa resulting from chemotherapy or radiotherapy for cancer treatment. This condition is primarily managed by synthetic compounds such as allopurinol, chlorhexidine, diphenhydramine, aluminum hydroxide, and palifermin. However, over time, the preference for herbal medicines has increased in terms of their lower interference with anticancer drugs and fewer side effects [ 41 ].

Aloe vera is one of the most well-known medicinal plants for treating and improving oral diseases. It contains active compounds such as polysaccharides, anthraquinones, vitamins C and E, and minerals, which have anti-inflammatory, antioxidant, and wound-healing properties [ 42 ]. Studies have shown that the use of aloe vera gel or mouthwash in patients undergoing radiotherapy helps reduce the severity of ulcers and alleviate pain [ 42 - 43 ]. For example, in a double-blind clinical trial involving 75 patients, those who rinsed their mouths with 5mL of aloe vera mouthwash experienced less severe mucositis compared to the placebo group from weeks 4 to 6. The mouthwashes were used for 2 minutes, three times daily, for six weeks in this research. However, no significant difference was observed between the two groups during the first three weeks. This study demonstrated the efficacy of aloe vera in improving oral mucositis in patients undergoing radiotherapy [ 43 ].

Chamomile, due to its content of chamazulene, alpha-bisabolol, and flavonoids, possesses anti-inflammatory, antibacterial, and pain-relieving properties. Studies have shown that this plant can reduce the healing time of oral ulcers, prevent secondary infections. Moreover, it is mentioned that its mouthwash form is as effective as allopurinol in reducing the severity of mucositis [ 42 , 44 ]. Supporting this matter, a review study by de Lima Dantas *et al*. [ 44 ] explained that topical chamomile at concentrations of 1% to 2.5%, used one to four times daily, was effective in preventing and treating oral mucositis in four out of six studies compared to allopurinol, showing positive results.

Green tea (*Camellia sinensis*) is rich in polyphenols and potent antioxidants such as epigallocatechin gallate, which reduce inflammation and strengthen oral mucosal cells. As a result, a mouthwash containing green tea extract has been shown to reduce the severity and duration of mucositis by up to 40% in cancer patients [ 42 ]. Additionally, studies indicate that green tea helps protect oral cells by reducing the production of free radicals. In one study, the intervention group rinsed their mouths with a solution of 5 grams of green tea in 100 mL of water for 60 seconds after brushing for six months. The control group rinsed their mouth with 100 mL of tap water for the same duration. The results showed that, at the end of the follow-up period, oral hygiene and mucosal membrane preservation were significantly improved in the intervention group compared to the control group, and the likelihood of developing mucositis was reduced [ 42 ].

Herbal interventions, particularly aloe vera, chamomile, and green tea, demonstrate significant efficacy in mitigating chemotherapy/radiotherapy-induced oral mucositis. Regarding this matter, some clinical studies confirm herbs' ability to reduce ulcer severity and accelerate healing while avoiding interference with anticancer therapies, offering a safer adjunct to conventional treatments [ 41 - 44 ].

### Recurrent Aphthous Stomatitis (RAS)

RAS is one of the most common oral diseases, characterized by recurrent and painful ulcers inside the mouth. Various factors, including genetic predisposition, immune disorders, microbial infections, psychological stress, and hormonal status, contribute to the development of RAS. Conventional treatments such as corticosteroids, antibiotics, and analgesics are typically used to control symptoms, but these can cause side effects and lead to drug resistance. As a result, the use of herbal medicines as an alternative option has gained attention [ 12 , 45 ].

*pomegranate extract* (*Punica granatum*), due to its anti-inflammatory and antioxidant properties, has been used as an effective treatment to reduce the healing time of oral ulcers in patients with RAS. In a clinical trial study, the average healing time of ulcers in the group using a 10% topical pomegranate gel three times daily for one week was significantly shorter than in the placebo group. This outcome indicated the notable efficacy of pomegranate in accelerating the healing of oral ulcers [ 12 ].

In addition to pomegranate, plants such as *Satureja khuzestanica* extract and the Chinese herbal formula *Fufangjiaolianzhiji* can be used as effective treatments for managing RAS due to their anti-inflammatory, antibacterial, and healing properties. However, further studies and clinical trials are needed to confirm these results and optimize dosage and administration methods [ 12 ].

To sum up, RAS can be effectively managed with herbal alternatives like *pomegranate extract*, which significantly reduces ulcer healing time through its anti-inflammatory and antioxidant properties. This is while other botanicals such as *Satureja khuzestanica* and *Fufangjiaolianzhiji* show promise but require further clinical validation to establish optimal dosing and efficacy as safer, side-effect-free alternatives [ 12 , 45 ].

### Oral Herpes

Oral herpes, also known as recurrent herpes simplex labialis, is primarily caused by the herpes simplex virus type 1. The duration of the disease typically ranges from 7 to 14 days, and common treatments such as acyclovir, valacyclovir, and famciclovir are often recommended. These drugs act as modified nucleosides and inhibit viral replication by targeting viral DNA polymerase, the primary enzyme responsible for viral replication [ 11 ].

In contrast to synthetic drugs, Gene-Eden-VIR/ Novirin has been introduced in studies as a promising herbal treatment for combating viral infections, particularly herpes simplex virus type 1 [ 11 , 28 ]. This formulation consists of five main plant-based ingredients: quercetin, green tea extract, cinnamon extract, licorice extract, and selenium. Each of these components has been selected for its antiviral, anti-inflammatory, and antioxidant properties, aiming to reduce viral load and enhance the immune system [ 26 ]. In a clinical study conducted by H. Polansky *et al*. [ 11 ], participants were administered between 1 to 4 capsules of Gene-Eden-VIR/ Novirin daily, with treatment durations varying from 2 months to 36 months. The results indicated that 89.3% of participants experienced a significant reduction in the frequency and duration of oral herpes outbreaks. Additionally, the intervention group reported fewer side effects compared to the placebo group, which used conventional antiviral synthetic drugs. Consequently, this treatment regimen, due to its flexible dosing and lower incidence of side effects, has been proposed as a promising alternative in this study [ 11 ].

Herbal formulations like Gene-Eden-VIR/Novirin demonstrate clinically significant efficacy against oral herpes, reducing outbreak frequency/duration in 89.3% of users with fewer side effects than conventional nucleoside analogs. These observations offer them as a promising immune-modulating alternative that targets viral replication while enhancing host defenses through synergistic antioxidant and anti-inflammatory actions [ 11 , 26 , 28 ].

### Oral Lichen Planus (OLP) 

OLP is a chronic inflammatory and potentially precancerous condition driven by T-cell-mediated immune dysregulation and an imbalance of pro-inflammatory cytokines (*e.g.*, TNF-α, IL-6), leading to keratinocyte apoptosis and mucosal damage. Triggers such as stress, infections, dental allergens, and oxidative stress contribute to its pathogenesis. The erythematous and ulcerative forms cause significant pain and burning, with a small risk of malignant transformation. While conventional treatments like corticosteroids and immunosuppressants are used, herbal agents offer a promising auxiliary treatment due to their anti-inflammatory, antioxidant, immunomodulatory, and analgesic properties [ 46 - 47 ]. 

Curcumin (*Curcuma longa*), the primary bioactive compound in turmeric, is a well-recognized natural polyphenol with potent antioxidant, anti-inflammatory, and anticancer properties which play a key role in the prevention and management of OLP [ 48 ]. Regarding this matter, Thomas *et al*. [ 49 ] studied the efficacy of curcumin on 75 patients diagnosed with OLP who were divided into three groups. These groups were defined as Group 1 (0.1% Triamcinolone acetonide oral paste thrice daily in tapering doses), Group 2 (1% curcumin oral gel thrice daily) and Group 3 (curcumin oral gel six times daily). The analysis of pain and signs of OLP after the intervention period showed that the treatment outcomes are better in Group 1 compared to Groups 2 and 3. Therefore, this study manifested that curcumin oral gel has shown potential in producing clinical improvements in patients with OLP; however, it is not suitable to be used as a primary treatment [ 49 ].

In another recent study, the efficacy of aloe vera in the management of OLP was evaluated. In this research, a total of 60 patients with OLP were divided into 2 groups. One group was administered 97% aloe vera gel topically along with 10 ml of 94.7% aloe vera juice taken orally twice daily. The active control group received 0.05% clobetasol propionate ointment applied topically twice a day. The treatment phase lasted for two months, followed by a four-month follow-up period. The outcomes of this study exhibited that clobetasol propionate is more effective than aloe vera for OLP management, but aloe vera can be a safe auxiliary treatment for OLP management [ 50 ]. As it is witnessed in the mentioned studies, it seems that herbal medicines cannot be used as a definitive alternative in OLP management [ 48 - 50 ].

### Burning Mouth Syndrome (BMS) 

BMS is a chronic and complex condition characterized by a persistent burning sensation or pain inside the mouth. This condition, which occurs without identifiable lesions or obvious causes, is most commonly observed in postmenopausal women aged 50 to 70. The uncertainty surrounding its etiology has made the diagnosis and management of BMS a significant challenge for clinicians. As a result, herbal treatments have been explored as complementary options for managing BMS [ 9 ].

Capsaicin, an active compound extracted from chili peppers, has been identified as an effective therapeutic option for alleviating BMS. Due to its analgesic properties and ability to reduce neural sensitivity, capsaicin is used topically to reduce localized pain [ 9 ]. A short-term clinical trial over two months demonstrated that the use of a topical capsaicin rinse (250mg of chili pepper powder in 50 ml of water at a concentration of 3.54µg/ml) led to a significant reduction in the visual analog scale pain score. At this stage, 76% of participants reported improvement in symptoms, with a statistically significant difference in pain relief compared to the placebo group. The same study further revealed that, over a long-term period of four months, the compound maintained its pain-reducing effects, although no statistically significant difference was observed compared to the placebo in some cases. By the end of the four months, 67% of patients reported a reduction in pain intensity, while some experienced minimal changes or worsening of pain. Importantly, no significant side effects were observed during the trial, highlighting capsaicin as a notable and effective therapeutic option for managing BMS. This review suggests that natural and herbal treatments can be beneficial in managing BMS symptoms. However, due to the complexity of its etiology and the variability of symptoms, further research is needed to determine the most effective treatment strategies [ 9 , 51 ].

### Halitosis

Halitosis is defined as the presence of an unpleasant odor in exhaled breath. Various causes have been proposed to explain its etiology, including intraoral factors such as dental caries, periodontal issues, and coated tongue. Meanwhile, extraoral factors such as gastrointestinal disorders are also defined as its etiological factors. Since halitosis is primarily caused by poor oral hygiene, common treatments include dental caries removal, scaling, and the use of toothbrushes and mouthwashes to clean the tongue [ 52 - 53 ]. Despite the high efficacy of existing mechanical methods in improving halitosis, herbal treatments have recently been proposed as complementary therapeutic options [ 51 ].

In a laboratory study, the effect of an oral spray containing Siwak (*Salvadora persica*) extract at concentrations of 1%, 1.5%, and 2% was evaluated against common caries-causing bacteria such as *Streptococcus mutans*. The study found that the 2% concentration, despite having a worse taste, color, and potential patient acceptance compared to the other concentrations, exhibited the highest antibacterial efficacy. However, after evaluating all parameters, the authors concluded that the 1% concentration, with its acceptable antibacterial effect and the best taste, odor, and color, was the most suitable formulation for clinical use [ 54 ].

Another widely used plant in oral hygiene improvement is licorice [ 55 ]. Licorice has been reported to exert powerful anti-inflammatory effects against inflammatory responses induced by lipopolysaccharide from periodontal bacteria such as *A. actinomycetemcomitans* and *P. gingivalis* [ 56 ]. Supporting these findings, a study by Meenakshi Rajendiran *et al*. [ 36 ] demonstrated that consuming licorice candy twice daily for three weeks significantly reduced the number of caries-causing bacteria. Additionally, the polysaccharide extract from its root showed strong anti-adhesive effects against *P. gingivalis*, a bacterium associated with the onset and progression of gingivitis and periodontitis.

Plants such as peppermint and green tea (*Camellia sinensis*) have also been shown to be effective in reducing dental plaque and bad breath due to their antibacterial and antioxidant properties. Peppermint, with its antibacterial properties, helps reduce bad breath, while green tea, through its catechins, improves gum health and reduces plaque formation [ 56 - 57 ]. In one study, patients who used toothpaste containing green tea extract with 60-90% epigallocatechin gallate for 2-5 minutes daily over four weeks experienced significant reductions in gingival inflammation and plaque formation [ 58 ].

Herbal interventions, including *Salvadora persica* (1% Siwak spray), licorice, peppermint, and green tea, demonstrate significant efficacy in managing halitosis. It can be concluded that these herbs offer synergistic benefits when combined with mechanical oral hygiene [ 52 - 58 ].

### Xerostomia

Xerostomia, or dry mouth, is a common oral condition caused by reduced salivary secretion. Given the wide range of underlying causes, various treatment approaches have been proposed to manage xerostomia. These treatments include stimulating saliva production through sugar-free gum or medications such as pilocarpine, bromhexine, and cevimeline, frequent water intake, and managing underlying diseases or medications causing dry mouth [ 59 ]. Due to the reported side effects of chemical treatments, herbal remedies have been suggested as alternatives for managing xerostomia [ 60 - 61 ].

In this context, a study by Zhongli Sun *et al*. [ 60 ] investigated the molecular mechanisms of *Mume Fructus*, a traditional Chinese medicine, in alleviating symptoms of Sjögren’s syndrome and dry mouth. The study identified 40 active compounds in this herbal medicine, among which quercetin, kaempferol, and β-sitosterol were notable for their efficacy in improving xerostomia. The findings suggested that *Mume Fructus* may modulate inflammation by targeting mediators such as AKT1 and IL-6, potentially preventing complications associated with Sjögren’s syndrome. However, further clinical research is needed to confirm its efficacy [ 60 ].

Following this matter, another study examined the effects of *Radix Paeoniae Alba*, another traditional Chinese herbal medicine, in improving dry mouth symptoms in patients with Sjögren’s syndrome. Key findings of this study indicated that β-sitosterol in *Radix Paeoniae Alba* increased salivary secretion, elevated intracellular calcium levels. These findings suggest that β-sitosterol could serve as a promising treatment for dry mouth in Sjögren’s syndrome patients, although more research is needed to confirm its long-term efficacy [ 61 ].

In addition to Chinese herbal medicines, other plants such as aloe vera, ginger, and cranberry juice have been proposed for managing dry mouth [ 62 ]. For example, a clinical trial by Pärnänen *et al*. [ 63 ] demonstrated that rinsing with 10 ml of cranberry mouthwash daily for 30 seconds over six months significantly improved both stimulated and unstimulated salivary secretion [ 63 ].

Similarly, a clinical trial by Badooei *et al*. [ 64 ] showed that using 20ml of 25% ginger mouthwash three times daily for two weeks significantly reduced symptoms of dry mouth compared to a placebo group using 50% aloe vera mouthwash. However, the study noted that both groups experienced improvements, suggesting that extracts from both plants can be used as complementary treatments for xerostomia [ 64 ]. Emerging herbal therapies, including *Mume Fructus*, *Radix Paeoniae Alba*, cranberry, ginger, and aloe vera, demonstrate promising efficacy in managing xerostomia. In this regard, clinical trials show significant improvements in both Sjögren’s syndrome and medication-induced dry mouth using herbal medicines, while avoiding the side effects of conventional sialagogues like pilocarpine [ 60 - 64 ].

In Table 1, a summary of the comparison between the effects of medicinal plants in treating common oral diseases and the gold standard treatment method is provided.

**Table 1:** Comparison of the Effects of Medicinal Plants with Common Treatment Methods for Oral Diseases

Herbal Medicine	Form and Concentration	Usage Instructions	Disease/ Condition	Gold Standard Treatment	Effects
Chinese flavonoid herbs [6]	-	Oral	Oral cancer	Surgery, radiotherapy, and chemotherapy	Inhibits cancer cell growth, activates caspases, and induces apoptosis
Strawberry [16]	-	Oral	Oral cancer	Surgery, radiotherapy, and chemotherapy	Reduces the risk of oral cells transforming into cancerous cells
Turmeric [16]	-	Oral	Oral cancer	Surgery, radiotherapy, and chemotherapy	Inhibits cell cycle, induces apoptosis
Triphala [17]	125, 250, 500, 1000, and 2000 µg/ml	Oral	Oral cancer	Surgery, radiotherapy, and chemotherapy	Reduces cancer cell proliferation, inhibits cell migration and invasion
Herbal paste [7]	10g of turmeric, tulsi powder, and honey	Apply paste 4 times daily for 3 months	Oral submucous fibrosis	Intralesional steroids	Improves mouth opening, reduces tongue protrusion, burning sensation, whitening of the mucous membrane, and palpable fibrous bands
Triphala [24]	5 µg/ml	Oral	Oral submucous fibrosis	Intralesional steroids	Reduces the negative effects of arecoline
Garlic [26]	Aqueous solution (40 mg/ml)	3-4 times daily for 4 weeks	Oral candidiasis	Nystatin, clotrimazole	Reduces symptoms of candidiasis without serious side effects
Thyme [29]	Spray (25 µl/ml)	8-10 sprays on denture	Oral candidiasis	Nystatin, clotrimazole	Reduces Candida albicans on acrylic resin dentures
Capsaicin [31]	High concentration (95%)	Mouthwash	Oral candidiasis	Nystatin, clotrimazole	Prevents Candida albicans hyphae formation and ergosterol biosynthesis
Licorice [26]	Oral extract (200 mg/kg)	Twice daily for 5 days	Oral candidiasis	Nystatin, clotrimazole	Reduces infection and Candida albicans levels
Cinnamon [26]	Mouthwash (635 µg/ml)	3 times daily for 15 days	Oral candidiasis	Nystatin, clotrimazole	Reduces symptoms of denture stomatitis
Berberine [26]	1.95 to 250 mg/L	Oral	Oral candidiasis	Miconazole, fluconazole	Stronger antifungal properties than s synthetic drugs
Ginger [26]	Mouthwash (0.625 to 5 mg/ml)	Various concentrations for 24 hours	Oral candidiasis	Nystatin, clotrimazole	Reduces Candida biofilm formation
Psidium guajava [37]	Gel containing 100 g of leaves	Apply gel to agar culture containing bacteria	Oral ulcers	Gel, tablets, mouthwash, patches, paste, and analgesic gum	Reduces ulcer healing time
Turmeric [38]	0.5 g gel containing Psidium guajava, turmeric, ethanol, and distilled water	Apply gel to Candida albicans culture	Oral ulcers	Gel, tablets, mouthwash, patches, paste, and analgesic gum	Reduces ulcer healing time
Aloe vera [39]	Gel containing 80% aloe vera	Apply twice daily with 15-minute massage	Traumatic oral ulcers	0.12% chlorhexidine gel	Reduces ulcer formation and healing time
Aloe vera [42]	Mouthwash	5 ml mouthwash for 2 minutes, 3 times daily for 6 weeks	Oral mucositis	Allopurinol, chlorhexidine, diphenhydramine, aluminum hydroxide, and palifermin	Reduces mucositis severity compared to placebo from weeks 4 to 6
Chamomile [43]	Topical gel (1% to 2.5%)	1 to 4 times daily	Oral mucositis	Allopurinol, chlorhexidine, diphenhydramine, aluminum hydroxide, and palifermin	Improves the prevention and treatment of oral mucositis
Green tea [41]	Solution (5 g green tea in 100 ml water)	Once daily for 60 seconds up to 6 months	Oral mucositis	Allopurinol, chlorhexidine, diphenhydramine, aluminum hydroxide, and palifermin	Improves oral hygiene, maintains the mucous membrane, and reduces mucositis risk
Pomegranate [11]	Topical gel (10%)	3 times daily for 1 week	Recurrent aphthous stomatitis	Corticosteroids, antibiotics, and analgesics	Reduces the healing time of oral ulcers
Gene-Eden-VIR/Novirin [10]	Capsule	1 to 4 capsules daily for 2 to 36 months	Oral herpes	Acyclovir, valacyclovir, famciclovir	Reduces the frequency and duration of herpes outbreaks with fewer side effects
Curcumin [49]	Topical gel (1%)	3 times and 6 times a day	Oral Lichen Planus	Corticosteroids and immunosuppressants	Reduces pain and signs of OLP of less than standard treatment
Aloe vera [50]	Topical gel (97%) along with 10 ml of 94.7% aloe vera juice	2 times daily	Oral Lichen Planus	Corticosteroids and immunosuppressants	Reduces pain and signs of OLP of less than standard treatment
Capsaicin [8]	250mg chili powder in 50ml water (3.54 µg/ml)	Gargle solution twice daily for 2 to 4 months	Burning mouth syndrome	Analgesics	Reduces pain and burning symptoms in 67% of patients after 4 months, with no significant side effects
Siwak [48]	Oral spray (1%)	-	Halitosis	Mechanical removal of caries	Reduces Streptococcus mutans levels
Licorice [35]	Licorice candy	Twice daily for 3 weeks	Halitosis	Mechanical removal of caries and scaling	Reduces oral bacteria and gingival inflammation
Green tea [57]	Toothpaste with green tea extract (60-90% EGCG)	2-5 minutes daily for 4 weeks	Halitosis	Scaling	Reduces plaque formation and gingival inflammation
Cranberry [62]	Mouthwash	10 ml daily for 30 seconds up to 6 months	Dry mouth	Saliva-stimulating drugs (e.g., pilocarpine, cevimeline)	Improves stimulated and unstimulated salivary secretion
Ginger [63]	Mouthwash (25%)	20 ml 3 times daily for 2 weeks	Dry mouth	Saliva-stimulating drugs (e.g., pilocarpine, cevimeline)	Improves symptoms of dry mouth
Aloe vera [63]	Mouthwash (50%)	20 ml 3 times daily for 2 weeks	Dry mouth	Saliva-stimulating drugs (e.g., pilocarpine, cevimeline)	Improves symptoms of dry mouth

### Limitations and challenges of using herbal medicines

While natural products offer significant potential benefits, their use in pharmaceuticals also presents certain challenges. These substances often consist of complex mixtures of compounds or extracts that produce diverse effects on biological systems. This matter requires a deeper understanding of their mechanisms of action. Nonetheless, researching natural products and phytochemicals is particularly challenging due to their inherent complexity. Moreover, the therapeutic efficacy of natural compounds may be influenced by the extraction, isolation, or purification processes. In other words, extraction methods involving heat, pH changes, or organic solvents can chemically alter sensitive phytochemicals, potentially diminishing their bioactivity. Additionally, factors such as geographic location, climate, and cultivation methods can influence the quality and potency of natural products. Therefore, the use of herbal medicine in oral treatments should be fully observed and managed [ 62 , 65 ].

## Conclusion

The integration of herbal medicine into dental treatments has demonstrated significant potential in improving specific clinical outcomes. These outcomes are defined as reduced gingival inflammation, enhanced management of recurrent aphthous ulcers, better alleviation of oral mucositis, and enhanced postoperative wound healing. Herbal agents like aloe vera, turmeric, green tea, and garlic have shown more notable antimicrobial, anti-inflammatory, and antioxidant properties, contributing to superior therapeutic effects compared to other herbs. Their favorable safety profiles and higher patient acceptability make them valuable adjuncts to conventional dental therapies. Nonetheless, the widespread clinical adoption of herbal treatments is hindered by issues such as inconsistent formulation quality, lack of dosage standardization, and insufficient data on long-term effects and herb-drug interactions. Therefore, future studies should prioritize well-designed randomized controlled trials, mechanistic investigations, and regulatory standardization efforts. With rigorous validation, herbal medicine may transition from a complementary option to an evidence-based component of mainstream dental practice, particularly for managing chronic and treatment-resistant oral conditions.

## References

[1] Amanpour S, Akbari Javar  M, Sarhadinejad Z, Doustmohammadi M, Moghadari M, Sarhadynejad Z ( 2023). A systematic review of medicinal plants and herbal products' effectiveness in oral health and dental cure with health promotion approach. J Educ Health Promot.

[2] Moghadam ET, Yazdanian M, Tahmasebi E, Tebyanian H, Ranjbar R, Yazdanian A, et al ( 2020). Current herbal medicine as an alternative treatment in dentistry: In vitro, in vivo and clinical studies. Eur J Pharmacol.

[3] Urban-Chmiel R, Marek A, Stępień-Pyśniak D, Wiecz-orek K, Dec M, Nowaczek A, et al ( 2022). Antibiotic resistance in bacteria: A review. Antibiotics.

[4] Budala DG, Martu MA, Maftei GA, Diaconu-Popa DA, Danila V, Luchian I ( 2023). The Role of Natural Compounds in Optimizing Contemporary Dental Treatment-Current Status and Future Trends. J Funct Biomater.

[5] Yadav P, Tandon S, Khurana C, Chopra M ( 2024). Herbs in dentistry. J Global Oral Health.

[6] Page MJ, McKenzie JE, Bossuyt PM, et al The PRISMA 2020 statement: an updated guideline for reporting systematic reviews. https://www.bmj.com/cont-ent/bmj/372/bmj.n71.full.pdf.

[7] Huang YC, Sung MY, Lin TK, Kuo CY, Hsu YC ( 2024). Chinese herbal medicine compound of flavonoids adjunctive treatment for oral cancer. J Formos Med Assoc.

[8] Mobeen S, Sv R, Jd S, Prakash R, DS, Swayampakula  H, et al ( 2023). A Novel Herbal Paste Formulation of Turmeric, Tulsi, and Honey for the Treatment of Oral Submucous Fibrosis. Cureus.

[9] Tan HL, Smith JG, Hoffmann J, Renton T ( 2022). A systematic review of treatment for patients with burning mouth syndrome. Cephalalgia.

[10] Rodrigues CF, Rodrigues ME, Henriques MCR ( 2019). Promising Alternative Therapeutics for Oral Candidiasis. Curr Med Chem.

[11] Polansky H, Javaherian A, Itzkovitz E ( 2018). Clinical trial of herbal treatment geneeden-VIR/novirin in oral herpes. J Evid Based Integr Med.

[12] Li CL, Huang HL, Wang WC, Hua H ( 2016). Efficacy and safety of topical herbal medicine treatment on recurrent aphthous stomatitis: a systemic review. Drug Des Devel Ther.

[13] Lugo-Flores MA, Quintero-Cabello KP, Palafox-Rivera P, Silva-Espinoza BA, Cruz-Valenzuela MR, Ortega-Ra-mirez LA, et al ( 2021). Plant-Derived Substances with Antibacterial, Antioxidant, and Flavoring Potential to Formulate Oral Health Care Products. Biomedicines.

[14] Dehghani Nazhvani A, Sarafraz N, Askari F, Heidari F, Razmkhah M ( 2020). Anti-Cancer Effects of Traditional Medicinal Herbs on Oral Squamous Cell Carcinoma. Asian Pac J Cancer Prev.

[15] Levi LE, Lalla RV ( 2018). Dental Treatment Planning for the Patient with Oral Cancer. Dent Clin North Am.

[16] Ben-Arie E, Lottering B, Inprasit C, Yip HT, Ho WC, Ton G, et al ( 2022). Traditional Chinese medicine use in patients with oral cancer: A retrospective longitudinal cohort study in Taiwan. Medicine (Baltimore).

[17] Panjwani S, Rai S, Misra D, Misra A ( 2016). Herbinaturals: A new paradigm in dentistry. J Indian Academy Oral Med Radi.

[18] Hu S, Li S, Xu Y, Huang X, Mai Z, Chen Y, et al ( 2024). The antitumor effects of herbal medicine Triphala on oral cancer by inactivating PI3K/Akt signaling pathway: based on the network pharmacology, molecular docking, in vitro and in vivo experimental validation. Phytomedicine.

[19] Kumar R, Mirza MA, Naseef PP, Kuruniyan MS, Zakir F, Aggarwal G ( 2022). Exploring the Potential of Natural Product-Based Nanomedicine for Maintaining Oral Health. Molecules.

[20] Dioguardi M, Ballini A, Sovereto D, Spirito F, Cazzolla AP, Aiuto R, et al ( 2022). Application of the Extracts of Punica granatum in Oral Cancer: Scoping Review. Dent J (Basel).

[21] Peng SY, Hsiao CC, Lan TH, Yen CY, Farooqi AA, Cheng CM, et al ( Toxicol 2020). Pomegranate extract inhibits migration and invasion of oral cancer cells by downregulating matrix metalloproteinase-2/9 and epithelial-mesenchymal transition. Environ.

[22] Gao W, Wang J, Zhao J ( 2022). Describing a modern chemotherapeutic drug prepared by Au nanoparticles to treat the human oral squamous cell carcinoma: A pre-clinical trial study. Inorg Chem Commun.

[23] Rajesh Kashyap R, Shanker Kashyap R ( 2021). Herbal derivatives in the management of mouth opening in oral sub mucous fibrosis-A network meta-analysis. Oral Dis.

[24] Shih YH, Wang TH, Shieh TM, Tseng YH ( 2019). Oral Submucous Fibrosis: A Review on Etiopathogenesis, Diagnosis, and Therapy. Int J Mol Sci.

[25] Patil S, Sarode SC, Ashi H, Ali Baeshen H, Thirumal Raj  A, Awan KH, et al ( 2021). Triphala extract negates arecoline-induced senescence in oral mucosal epithelial cells in vitro. Saudi J Biol Sci.

[26] Černáková L, Rodrigues CF ( 2020). Microbial interactions and immunity response in oral Candida species. Future Microbiol.

[27] Gharibpour F, Shirban F, Bagherniya M, Nosouhian M, Sathyapalan T, Sahebkar A ( 2021). The Effects of Nutraceuticals and Herbal Medicine on Candida albicans in Oral Candidiasis: A Comprehensive Review. Adv Exp Med Biol.

[28] Mosaddad SA, Hussain A, Tebyaniyan H ( 2023). Green alternatives as antimicrobial agents in mitigating periodontal diseases: A narrative review. Microorganisms.

[29] Shafaroudi AM, Gorji NE, Nasiri P, Javidnia J, Saravi ME ( 2022). Antifungal properties of zataria multiflora on candida species: A systematic review. J Evid Based Integr Med.

[30] Aghajani A, Abastabar M, Khalilian A, Haghani I, Jannati P, Ebrahimisaravi M ( 2019). Comparing the antifungal effect of Sodium Hypochlorite, Deconex, Zataria multiflora, and Artemisia aucheri on the surface of acrylic resin dentures: An experimental study. J Maz Uni Med Sci.

[31] Zhang W, Zhang Y, Fan J, Feng Z, Song X ( 2024). Pharmacological activity of capsaicin: Mechanisms and controversies (Review). Mol Med Rep.

[32] Behbehani JM, Irshad M, Shreaz S, Karched M ( 2023). Anticandidal Activity of Capsaicin and Its Effect on Ergosterol Biosynthesis and Membrane Integrity of Candida albicans. Int J Mol Sci.

[33] Eid Abdelmagyd HA, Ram Shetty DS, Musa Musleh Al-Ahmari  DM ( 2019). Herbal medicine as adjunct in periodontal therapies- A review of clinical trials in past decade. J Oral Biol Craniofac Res.

[34] Khan SF, Shetty B, Fazal I, Khan AM, Mir FM, Moothedath M, et al ( 2023). Licorice as a herbal extract in periodontal therapy. Drug Target Insights.

[35] AlDehlawi H, Jazzar A ( 2023). The Power of Licorice (Radix glycyrrhizae) to Improve Oral Health: A Comprehensive Review of Its Pharmacological Properties and Clinical Implications. Healthcare (Basel).

[36] Rajendiran M, Trivedi HM, Chen D, Gajendrareddy P, Chen L ( 2021). Recent Development of Active Ingredients in Mouthwashes and Toothpastes for Periodontal Diseases. Molecules.

[37] Shahare N, CHOUHAN S, Darwhekar GN ( 2021). Herbs used in treatment of mouth ulcer- a review: Herbal medicines for treatment of mouth ulcer. Int J Pharm Chem.

[38] Bandana K, Kumar S, Sagar MK, Tyagi H ( 2020). Formulation and evaluation of herbal gel of guava leaves and liquorice roots extract for using mouth ulcer. J Crit Rev.

[39] Sing R, Bansal S, Kumar Mishra  M ( 2020). Formulation and evaluation of herbal oral gel containing extracts of powdered Psidium guajava linn leaves with Curcuma longa linn rhizomes to treat mouth ulcer. Int J Drug Develop Res.

[40] Leiva-Cala C, Lorenzo-Pouso AI, Centenera-Centenera B, López-Palafox J, Gándara-Vila P, García-García A, et al ( 2020). Clinical efficacy of an Aloe Vera gel versus a 0.12% c-hlorhexidine gel in preventing traumatic ulcers in patients with fixed orthodontic appliances: a double-blind randomized clinical trial. Odontology.

[41] Baharvand M, Jafari S, Mortazavi H ( 2017). Herbs in Oral Mucositis. J Clin Diagn Res.

[42] Ferreira AS, Macedo C, Silva AM, Delerue-Matos C, Costa P, Rodrigues F ( 2022). Natural products for the prevention and treatment of oral Mucositis: A review. Int J Mol Sci.

[43] Najafizade N, Mobini Dehkordi R, Hemati S ( 2024). Investigating the effect of Aloe vera on the prevention and treatment of radiotherapy-induced oral mucositis in patients with head-and-neck cancer. J Res Med Sci.

[44] De Lima Dantas JB, Freire TFC, Sanches ACB, Julião ELD, Medrado ARAP, Martins GB ( 2022). Action of Matricariarecutita (chamomile) in the management of radiochemotherapy oral mucositis: A systematic review. Phytother Res.

[45] Shavakhi M, Sahebkar A, Shirban F, Bagherniya M ( 2022). The efficacy of herbal medicine in the treatment of recurrent aphthous stomatitis: A systematic review of randomized clinical trials. Phytother Res.

[46] Ghahremanlo A, Boroumand N, Ghazvini K, Hashemy SI ( 2019). Herbal medicine in oral lichen planus. Phytother Res.

[47] Pourshahidi S, Sheykhbahaei N ( 2021). Effectiveness of herbal based medications in the treatment of oral lichen planus: a review article. J Herb Med.

[48] Kalaskar AR, Bhowate RR, Kalaskar RR, Walde SR, Ramteke RD, Banode PP ( 2020). Efficacy of Herbal Interventions in Oral Lichen Planus: A Systematic Review. Contemp Clin Dent.

[49] Thomas AE, Varma B, Kurup S, Jose R, Chandy ML, Kumar SP, et al ( 2017). Evaluation of efficacy of 1% Curcuminoids as local application in management of oral lichen planus- interventional study. J Clin Diagn Res.

[50] Vaidya A, Khorate M, Chinam N, Figueiredo N ( 2023). Efficacy of Aloe Vera and Clobetasol Propionate in the Management of Oral Lichen Planus: A Randomized Parallel Clinical Trial. Front Dent.

[51] Rehman S, Iqbal Z, Qureshi R, AlOmar TS, Almasoud N, Younas M, et al ( 2024). Ethno-Dentistry of Medicinal Plants Used in North Waziristan, Pakistan. Int Dent J.

[52] Khounganian RM, Alasmari ON, Aldosari MM ( 2023). Causes and management of halitosis: A narrative review. Cureus.

[53] Wu J, Cannon RD, Ji P, Farella M, Mei L ( 2020). Halitosis: prevalence, risk factors, sources, measurement and treatment: a review of the literature. Aust Dent J.

[54] Resmisari R, Wicaksono S, Alfiani N, Effendi S ( 2021). Siwak (Salvadora persica) extract as a natural anti-halitosis mouth spray. IOP Conf Se Earth Environ Sci.

[55] Forouzanfar A, Mohammadipour HS, Forouzanfar F ( 2021). The Potential Role of Tea in Periodontal Therapy: An Updated Review. Curr Drug Discov Technol.

[56] Henley-Smith CJ, Kok AM, Botha FS, Baker C, Lall N ( 2024). The effect of a poly-herbal plant extract on the adhesion of Streptococcus mutans to tooth enamel. BMC Complement Med Ther.

[57] Pasupuleti MK, Nagate RR, Alqahtani SM, Penmetsa GS, Gottumukkala S, Ramesh KSV ( 2023). Role of Medicinal Herbs in Periodontal Therapy: A Systematic Review. J Int Soc Prev Community Dent.

[58] Basu A, Masek E, Ebersole JL ( 2018). Dietary Polyphenols and Periodontitis-A Mini-Review of Literature. Molecules.

[59] Mhatre S, Srichand R, Sethumadhavan J, Mishra PB, Patil SD, Chavan RS, et al ( 2024). Dry Mouth Dilemma: A Comprehensive Review of Xerostomia in Complete Denture Wearers. Cureus.

[60] Sun Z, Deng L, Xu Z, Yang K, Yu P ( 2024). Uncovering the molecular mechanism of Mume Fructus in treatment of Sjögren's syndrome. Medicine (Baltimore).

[61] Wu F, Wu G, Li T, Lu W, Fu T, Zhang Z ( 2023). Exploring the target and mechanism of radix paeoniae Alba on Sjogren's Syndrome. Comb Chem High Throughput Screen.

[62] Kontogiannopoulos KN, Kapourani A, Gkougkourelas I, Anagnostaki ME, Tsalikis L, Assimopoulou AN, et al ( 2023). A Review of the Role of Natural Products as Treatment Approaches for Xerostomia. Pharmaceuticals (Basel).

[63] Pärnänen P, Lomu S, Räisänen IT, Tervahartiala T, Sorsa T ( 2022). Effects of Fermented Lingonberry Juice Mouthwash on Salivary Parameters-A One-Year Prospective Human Intervention Study. Dent J (Basel).

[64] Badooei F, Imani E, Hosseini-Teshnizi S, Banar M, Memarzade M ( 2021). Comparison of the effect of ginger and aloe vera mouthwashes on xerostomia in patients with type 2 diabetes: A clinical trial, triple-blind. Med Oral Patol Oral Cir Bucal.

[65] Dzobo K (2022). The role of natural products as sources of therapeutic agents for innovative drug discovery. Comprehensive Pharmacology.

